# Understanding HIV Risk Behavior among Tuberculosis Patients with Alcohol Use Disorders in Tomsk, Russian Federation

**DOI:** 10.1371/journal.pone.0148910

**Published:** 2016-02-12

**Authors:** Ann C. Miller, A. Katrina Nelson, Viktoria Livchits, Shelly F. Greenfield, Galina Yanova, Sergei Yanov, Hilary S. Connery, Sidney Atwood, Charmaine S. Lastimoso, Sonya S. Shin, on behalf of the Tomsk Tuberculosis Alcohol Working Group

**Affiliations:** 1 Department of Global Health and Social Medicine, Harvard Medical School, Boston, MA, United States of America; 2 Division of Global Health Equity, Brigham and Women’s Hospital, Boston, MA, United States of America; 3 Partners In Health Representative Office in Russian Federation, Moscow, Russia; 4 Alcohol and Drug Abuse Treatment Program, McLean Hospital, Belmont, MA United States of America; 5 Department of Psychiatry, Harvard Medical School, Boston, MA, United States of America; 6 Division of Women's Mental Health, McLean Hospital, Belmont, MA, United States of America; 7 Tomsk Oblast Tuberculosis Hospital, Tomsk, Russian Federation; Public Health Agency of Barcelona, SPAIN

## Abstract

Russian Federation’s (RF) HIV epidemic is the fastest growing of any country. This study explores factors associated with high HIV risk behavior in tuberculosis (TB) patients with alcohol use disorders in Tomsk, RF. This analysis was nested within the Integrated Management of Physician-delivered Alcohol Care for TB Patients (IMPACT, trial number NCT00675961) randomized controlled study of integrating alcohol treatment into TB treatment in Tomsk. Demographics, HIV risk behavior (defined as participant report of high-risk intravenous drug use and/or multiple sexual partners with inconsistent condom use in the last six months), clinical data, alcohol use, depression and psychosocial factors were collected from 196 participants (161 male and 35 female) at baseline. Forty-six participants (23.5%) endorsed HIV risk behavior at baseline. Incarceration history(Odds Ratio (OR)3.93, 95% confidence interval (CI) 1.95, 7.95), age under 41 (OR:2.97, CI:1.46, 6.04), drug addiction(OR: 3.60 CI:1.10, 11.77), history of a sexually transmitted disease(STD)(OR 2.00 CI:1.02, 3.90), low social capital (OR:2.81 CI:0.99, 8.03) and heavier alcohol use (OR:2.56 CI: 1.02, 6.46) were significantly more likely to be associated with HIV risk behavior at baseline. In adjusted analysis, age under 41(OR: 4.93, CI: 2.10, 11.58), incarceration history(OR: 3.56 CI:1.55, 8.17) and STD history (OR: 3.48, CI: 1.5, 8.10) continued to be significantly associated with HIV risk behavior. Understanding HIV transmission dynamics in Russia remains an urgent priority to inform strategies to address the epidemic. Larger studies addressing sex differences in risks and barriers to protective behavior are needed.

## Introduction

In many settings where the HIV epidemic is driven by sexual transmission, alcohol use is an important risk factor. Research in many populations has linked alcohol use with HIV transmission via several mediating mechanisms, including psychosocial and socio-economic factors, as well as behavioral and biological risk factors. Underlying socio-economic vulnerability and instability are driving forces behind the convergence of alcohol use disorders (AUDs) and HIV, disproportionately affecting marginalized populations, such as the unemployed and incarcerated; the same populations also at increased risk for tuberculosis (TB). Heavy alcohol use affects high-risk sexual behavior in a number of ways, including its association with lower levels of condom use and/or multiple sex partners. This association has been demonstrated in diverse populations such as gay men [1] and men who have sex with men in Moscow, Russia, [2] adolescents, [3] men and women in East and Southern Africa, [4] and people with HIV in the Russian Federation. [5] Heavy alcohol use is also associated with immunomodulation and impaired host defense, which may increase the risk of infection and disease progression among individuals exposed to HIV or tuberculosis. Heavy alcohol use has also been associated with decreased adherence to TB and HIV treatment, [6] reduced viral load suppression [6] and death in HIV-infected patients [7] (particularly in women) and TB patients. [8] Conversely, HIV diagnosis may also influence alcohol use: in a Russian study from 1996, diagnosis with HIV was associated with a later diagnosis of an alcohol use disorder in 22% of the study’s participants. [9]

The epidemic in the Russian Federation presents a critical challenge to HIV control: it is the fastest growing HIV epidemic of any country [10]. Russia experienced dramatic social transformation since the collapse of the communist state with subsequent soaring rates of crime, incarceration, and poverty. These conditions, combined with a weakened healthcare infrastructure, resulted in unprecedented health crises in the 1990s, including high rates of alcohol consumption and a precipitous drop in life expectancy, largely due to excessive alcohol use [11]. Although the HIV epidemic in Russia had been initially described as attributable to intravenous drug use, [10, 12] data from 2011 show that 43.4% of new cases were due to heterosexual transmission. [13] Additionally, studies have associated alcohol consumption with HIV risk behavior [14] and suggest that certain key populations at higher risk are particularly vulnerable to the negative effects of alcohol use and HIV risk behavior, such as individuals with tuberculosis. [15] Yet the intersection between heavy alcohol use and sexual HIV risk behavior among individuals living in the RF has until recently remained largely unexplored.

One strategy to reduce alcohol-associated HIV risk is to target high-risk populations that are engaged in care; the 2008 PREVENT study of an intervention in narcology clinics in Russia is one example. [16] Given high rates of both HIV and alcohol use among TB patients, this population may represent one such important risk-group in Russia and elsewhere. Furthermore, TB treatment (requiring daily administration of medications for months) provides an opportunity to deliver interventions to reduce HIV risk behavior and alcohol consumption. For this reason, we sought to understand the complex interactions between alcohol use and HIV risk among TB patients in Russia. This study explores factors associated with HIV risk behavior in men and women with alcohol use disorders in a population of tuberculosis patients in Tomsk, Russian Federation.

## Methods

### Setting and population

This study was nested within a randomized controlled study, Integrated Management of Physician-delivered Alcohol Care for Tuberculosis Patients (IMPACT) in Tomsk, located in Western Siberia (registered at clinicaltrials.gov, trial number NCT00675961). The study setting is described in detail elsewhere. [17, 18] To summarize, the IMPACT study was conducted among tuberculosis patients with alcohol use disorders (defined as any lifetime diagnosis of alcohol abuse or dependence based on the Diagnostic and Statistical Manual, 4^th^ edition (DSM-IV) as measured by the Composite International Diagnostic Interview-Substance Abuse Module (CIDI-SAM)[19]) receiving care in a tuberculosis (TB) hospital in Tomsk Oblast, Russia, to assess the feasibility and effectiveness of integrating two modalities of alcohol treatment into the tuberculosis treatment program. [20] Tomsk Oblast has a population of 1,200,000, and, as of 2013, a tuberculosis incidence rate of 57.6 per 100,000, HIV incidence of 189.8 per 100,000 and TB/HIV prevalence of 13.4 per 100,000. [personal communication, A. Solovyova] No reliable data exist that describe alcohol use in Tomsk; however, one study found that 52% of total mortality among the 15–54 year old population in Tomsk is attributable to alcohol use. [21] Tuberculosis care in Tomsk is provided at no cost to patients and conducted under directly observed therapy. TB patients are screened for HIV and alcohol use disorders prior to initiation of TB therapy.

Ethics review and approval for the IMPACT study was provided by the Partners Healthcare Institutional Review Board; the State Research Center Virology and Biotechnology, Novosibirsk Region; and the Siberian State Medical University of the Federal Agency for Health Care and Social Development, Tomsk, Russia.

### Data collection

Screening interviews were conducted on all adults starting TB treatment at the TB Hospital. The Alcohol Use Disorders Identification Test (AUDIT) [22] was routinely used by TB physicians to assess alcohol use severity [17]. Patients meeting eligibility criteria—TB patients being treated at one of the 3 study sites with a concurrent alcohol use disorder as defined by the CIDI-SAM [19]–were approached for study enrollment. After signing informed consent, study participants were randomized to one of four arms (behavioral intervention, naltrexone, both, or treatment as usual) and followed for 6 months. Baseline data on demographics, clinical history and course, alcohol use, depression and psychosocial factors were collected through interviews and chart review. Baseline depression was assessed using the Center for Epidemiologic Studies Depression (CES-D) scale [23]. Alcohol use and HIV risk behavior were assessed with the Timeline Follow-Back Calendar (TLFB)[24] and a modified Risk Assessment Behavior (RAB) tool, [25] respectively. The Addiction Severity Index Scale (ASI)[26] was also applied to assess psychosocial consequences of alcohol use.

### Data analysis

The primary outcome for this analysis was baseline “HIV risk behavior,” defined as participant report of at least one of the following behaviors in the past six months: 1) high-risk injection drug use (IDU) and/or 2) multiple sexual partners with inconsistent condom use. Inconsistent condom use was defined as participant report that condoms had not been used every time the participant had sex. High-risk injection drug use was defined as injecting drugs and sharing or trading injection apparatus or supplies, or injecting drugs in the context of a shooting gallery. “Depression” was defined as a CES-D score of 16 or higher. Alcohol use severity was defined using five criteria: 1) number of drinking days (defined as a day in which any alcohol was consumed) in past month; 2) number of heavy drinking days (defined using US National Institute on Alcohol Abuse and Alcoholism standard as a day in which 4 or more standard drinks were consumed if female or 5 or more standard drinks were consumed if male) in the past month; 3) average number of drinks per drinking day; 4) average number of drinks per heavy drinking day; and 5) “zapoi” (a Russian term for extended binge drinking), defined as any episode of at least three consecutive heavy drinking days. Social capital was assessed based on a series of questions in a baseline questionnaire addressing trust in other people and ability to turn to family or friends in time of need. A social capital score was created by totaling the responses of these questions, ranging from 0 (least trust, fewest people to turn to in time of need, least resources) to 9 (most trust in others, most people, most resources) “Low social capital” was defined as an overall social capital score less than 4.

Data were analyzed in Stata 11.2 (College Park, TX). Chi squared tests and Fisher’s exact tests were used to assess associations between HIV risk behavior and binary or categorical variables, and t-tests or Wilcoxon rank-sum tests used for continuous data. Odds ratios were calculated using single and multiple logistic regression or exact logistic regression (using conditional maximum likelihood estimates for small numbers) to assess baseline data associations. Variables significant at *p* = 0.1 were considered for inclusion in the multivariable analysis. Alcohol use variables were tested for collinearity before entry into the model. A final model was determined using likelihood ratio testing. Variables included in the multivariable analysis were assessed for interaction and effect modification.

## Results

Two hundred patients were enrolled in the randomized controlled trial. [20] Of these 200, four patients were withdrawn from the study when active TB was ruled out; thus, 196 participants (82.1% males) comprised the cohort for this study. Demographic and clinical data for the participants are presented by gender in Table 1. Assessment of gender differences in substance use are presented in Table 2. Of the 196 participants, 46 (23.5%) endorsed HIV risk behavior at baseline. Five (2.6%) reported only risk behavior related to IDU, 37 (18.9%) reported only sex risk, and four (2.0%) reported both. Ninety-nine (50.5%) participants reported at least one heavy drinking day in the month before starting TB treatment, and ninety-seven (49.5%) reported at least one bout of “zapoi” [data not shown]. A higher proportion of men than women reported HIV risk behavior (26.1% vs. 11.4%, borderline statistically significant at p = 0.06). There were no gender differences in reported alcohol use including number of drinking days, binge drinking episodes or drinks per drinking day. No women reported injection drug risk behavior in the six months prior to the study.

**Table 1:** Socio-demographic characteristics of study participants, by sex. Tomsk, Russian Federation. n = 196.

Factors (n, if not 196)	Total	Male, N = 161 Mean [std] or n (%)	Female, N = 35 Mean [std] or n (%)
Age (n = 195)	41.5 [10.9]	42 [10.9]	39.1 [10.9]
Under 41 years (n = 195)	95(48.7)	77 (48.1)	18 (51.4)
Married or living together	74 (37.7)	59 (36.6)	15 (42.9)
Prior incarceration	53 (27.3)	51 (32.8)	2 (5.7)
Low social capital	16 (8.2)	12 (7.5)	4 (11.4)
Depression	26 (13.3)	17 (10.6)	9 (25.7)
History of sexually transmitted infection	73(37.2)	58(36.0)	15(42.9)
Current tobacco use	182 (92.8)	151 (93.8)	31 (88.6)
Low body mass index	56(28.6)	34(21.1)	22(62.9)
Multi-drug resistant tuberculosis	26(13.3)	22(13.7)	4(11.4)
HIV positive at intake	1(0.5)	0	1(2.9)

**Table 2:** Substance use and HIV risk characteristics of study participants, by sex. Tomsk, Russian Federation. n = 196.

Factors (n, if not 196)	Total	Male, N = 161 Mean [std] or n (%)	Female, N = 35 Mean [std] or n (%)	P-value
High Alcohol Severity Index (ASI) score	22 (11.2)	18 (11.0)	4 (11.4)	0.95
Heavy drinking days in 30 days prior to baseline	3.07 [5.2]	3.07 [5.2]	3.06 [5.5]	0.64
Mean # drinks per drinking day (n = 129) in 30 days prior to baseline	10.3(7.99)[8]	10.4(7.4)[8.9]	10.1(10.4)[5.4]	0.34**
Mean # drinks per heavy drinking day (n = 99) in 30 days prior to baseline	14.1[7.5]	14.04 [6.85]	14.2[10.32]	0.64**
Number of binge drinking (zapoi) episodes (n = 193) in 30 days prior to baseline	1.69 [2.5]	1.61 [2.3]	2.08 [3.1]	0.69**
AUDIT score (n = 194)	18.3 [7.7]	18.8 [7.7]	15.79 [7.07]	0.05
HIV risk behavior in the 6 months prior to baseline data collection	46 (23.5%)	42 (26.1)	4 (11.4)	0.06*
HIV risk components				
Drug risk only	5(2.6)	5(3.1)	0	
Sex risk only	37(18.9)	33(20.5)	4(11.4)	0.39*
Both	4(2.0)	4(2.5)	0	

* Fisher’s exact test

** Wilcoxon rank sum

We assessed associations between demographic and clinical characteristics and HIV risk behavior at baseline (Table 3). In unadjusted analysis, having a stable partner (married or living with a partner), a low body mass index (a possible proxy for illness severity) and greater number of days of alcohol abstinence in the month prior to diagnosis were significantly associated with a lower likelihood of HIV risk behavior. Incarceration history, younger age (40 or younger), a diagnosis of drug addiction, prior history of a sexually transmitted disease, low social capital and heavier alcohol use (in the form of higher AUDIT score and high ASI score) were significantly more likely to be associated with HIV risk behavior. In adjusted analysis, age, history of prior incarceration and history of a sexually transmitted disease continued to be significantly associated with HIV risk behavior.

**Table 3:** Factors associated with HIV high risk behavior at baseline among study participants, Tomsk, Russian Federation (n = 196).

Factors (n if not 196)	OR 95% CI	p-value	Adjusted OR 95% CI	P-value
Male sex*	2.73 (0.91, 8.21)	0.07	12.11 (1.38, 106.21)	0.02
Married or living together*	0.27 (0.12, 0.61)	<0.01	5.7 (0.48, 68.07)	0.17
Age less than 41 years*	2.97 (1.46, 6.04)	<0.01	4.93 (2.10, 11.58)	<0.001
Prior incarceration*	3.93 (1.95, 7.95)	<0.01	3.56 (1.55, 8.17)	<0.002
Low social capital*	2.81 (0.99, 8.03)	0.05		
Depression	1.23 (0.48, 3.15)	0.65		
History of sexually transmitted infection*	2.00 (1.02, 3.90)	0.04	3.48 (1.50, 8.10)	<0.01
Current tobacco use**	0.75 (0.20, 3.45)	0.85		
Low body mass index*	0.37 (0.15, 0.88)	0.03		
Multi-drug resistant tuberculosis	1.54(0.62, 3.82)	0.35		
Lifetime alcohol dependence disorder	1.18 (0.59, 2.35)	0.63		
Lifetime alcohol abuse disorder**	5.01(0.79, inf)	0.09		
Lifetime drug use disorder, DSM-IV *	3.60 (1.10, 11.77)	0.03		
High Alcohol Severity Index (ASI) score*	2.56 (1.02, 6.46)	0.046		
Heavy drinking days *	1.05 (0.99, 1.12)	0.07		
Abstinent days*	0.96 (0.93, 99)	0.03		
Mean # drinks per drinking day (n = 131) in 30 days prior to baseline	1.00 (99, 1.02)	0.33		
Mean # drinks per heavy drinking day (n = 107) in 30 days prior to baseline	1.00 (99, 1.02)	0.33		
AUDIT score *	1.05 (1.01, 1.10)	0.02		

*included into main effects multivariable model

**exact Logistic regression

## Discussion

This cohort of patients with tuberculosis and alcohol use disorders presents with great psychosocial vulnerabilities, including high levels of unemployment and past incarceration at the time of enrollment. Seventy-two percent of patients consecutively starting TB treatment were found to meet DSM-IV criteria for a history of alcohol abuse or dependence. Alcohol consumption in this cohort was high among both men and women, as reflected by the average ten drinks per drinking day at baseline in both genders. Although women’s alcohol consumption in Russia is known to be high (12.6 liters per capita in 2010)[27], this finding of gender parity for alcohol use in tuberculosis patients is striking and significant, as studies across many countries have found that women historically report lower levels of alcohol use than men, [28] including both WHO data [27] and a prospective study of alcohol and mortality in Russia. [29] However, these results are consistent with an earlier study in a similar population that showed that among people with AUDs in Tomsk, consumption among the women was not significantly less than that of men. [18] These high levels of alcohol consumption among female tuberculosis patients are notable and worth greater exploration for both their clinical and public health significance.

RAB scores in general were quite low, ranging from 0 to 17 out of a possible 40, consistent with other studies among Russian TB patients. [30] However, a high proportion (23.2%) practiced behaviors associated with high HIV risk in the six months prior to starting TB treatment, primarily the combination of inconsistent condom use (68% of the cohort) and multiple partners (26% of the cohort).

Alcohol use severity was associated with HIV risk behavior in unadjusted analysis, but lost statistical significance when adjusting for demographic and social factors. There are many possible explanations for this finding, particularly among a cohort of individuals with severe and long-standing alcohol use disorders, in which specific patterns of alcohol use may not have significantly influenced their degree of HIV risk behavior. For example, Ehrenstein et al demonstrated that in a cohort of HIV-infected people with alcohol problems, risky alcohol use (defined as >14 drinks per week in men or >7 in women) was associated with inconsistent condom use in active drug users but not in people who didn’t actively use injection drugs. [31] More recently, Wirtz et al report that in a Russian cohort of men who have sex with men, consistent condom use during anal sex was statistically significantly lower for heavy alcohol users than low–level users.[2] While heavy alcohol use has been associated with general increases in sexual HIV risk behavior in many settings at the global level, studies have shown a lack of association between alcohol use and condom use at the level of actual sexual event; one theory proposed is that one is either an habitual condom user or not, and alcohol does not change that behavior materially. [32] In addition, binge drinking may pose a different risk for risky sexual behaviors than chronic alcohol use. [33, 34]

It is also possible that we were unable to accurately measure HIV risk behavior. While the RAB is a useful “broad brush” to risk assessment, it was initially developed for and validated specifically with injection drug users. [35] Although the RAB is quite sensitive to both drug risk and sex risks for men who have sex with men, it may be less sensitive in exclusively heterosexual men and women. The RAB asks whether condoms had been used “all of the time, most of the time, some of the time, none of the time” but does not ask with which partners condoms were used. If a person in a relationship never used condoms with his/her spouse, but used them with others, this may overestimate true risk. Other, more precise tools to assess HIV risk factors in these groups may provide better estimates of true risk.

Two main limitations of the study are the small sample size and that it is based on secondary analysis of data from a clinical trial. Our small sample size impeded our ability to explore additional gender differences in HIV risk behavior; thus, we were unable to compare factors associated with risky behavior by gender, which is known to be associated in many studies with both sexual risk [36, 37] and alcohol risk behavior. [28] Gender differences in self-report of both sex and alcohol behaviors may also exist. Some studies have documented that women face more stigma with respect to alcohol use, [38] and studies specifically in Russia have documented women underreporting alcohol use. [39] We acknowledge that findings described in this cohort of TB patients are not generalizable to the broader population; nonetheless they could inform prevention and risk reduction interventions directed at such individuals while receiving TB treatment.

As the Russian HIV epidemic continues to accelerate and the alcohol use epidemic does not show signs of slowing, understanding the complexities of HIV transmission dynamics in Russia remains an urgent priority to inform strategies to address the epidemic. Studies addressing event-level HIV risk behaviors and qualitative studies on risky behavior, particularly aimed at understanding the complex relationship between alcohol use, infectious disease, gender, and other psychosocial factors in Russia are important in designing culturally relevant intervention strategies that target specific behavior such as condom use among vulnerable populations. Larger studies, specifically addressing sex differences in risks and barriers to protective behavior, would also be important.

## Supporting Information

S1 File IMPACT Aim 3 public dataset. (CSV)

S2 File Impact Aim 3 data dictionary. (LOG)

## References

[1] Penkower L, Dew MA, Kingsley L, Becker JT, Satz P, Schaerf FW, et al Behavioral, health and psychosocial factors and risk for HIV infection among sexually active homosexual men: the Multicenter AIDS Cohort Study. Am J Public Health. 1991;81(2):194–6. Epub 1991/02/01. 1990857 10.2105/ajph.81.2.194 PMC1404964

[2] Wirtz AL, Zelaya CE, Latkin C, Stall R, Peryshkina A, Galai N, et al Alcohol Use and Associated Sexual and Substance Use Behaviors among Men who Have Sex with Men in Moscow, Russia AIDS Behav. 2015 Epub 18 April 2015. 10.1007/s10461-015-1066-2. PubMed Central PMCID: PMC25893659. PMC4609587 25893659

[3] Cooper ML. Alcohol use and risky sexual behavior among college students and youth: evaluating the evidence. J Stud Alcohol Suppl. 2002;(14):101–17. Epub 2002/05/23. . 12022716 10.15288/jsas.2002.s14.101

[4] Kalichman SC, Simbayi LC, Kaufman M, Cain D, Jooste S. Alcohol use and sexual risks for HIV/AIDS in sub-Saharan Africa: systematic review of empirical findings. Prev Sci. 2007;8(2):141–51. Epub 2007/02/01. 10.1007/s11121-006-0061-2 . 17265194

[5] Krupitsky EM, Horton NJ, Williams EC, Lioznov D, Kuznetsova M, Zvartau E, et al Alcohol use and HIV risk behaviors among HIV-infected hospitalized patients in St. Petersburg, Russia. Drug Alcohol Depend. 2005;79(2):251–6. Epub 2005/07/09. S0376-8716(05)00054-2 [pii] 10.1016/j.drugalcdep.2005.01.015 16002034 PMC1360173

[6] Chander G, Lau B, Moore RD. Hazardous alcohol use: a risk factor for non-adherence and lack of suppression in HIV infection. J Acquir Immune Defic Syndr. 2006;43(4):411–7. Epub 2006/11/14. 00126334-200612010-00005 [pii]. 17099312 10.1097/01.qai.0000243121.44659.a4 PMC2704473

[7] Hessol NA, Kalinowski A, Benning L, Mullen J, Young M, Palella F, et al Mortality among participants in the Multicenter AIDS Cohort Study and the Women's Interagency HIV Study. Clin Infect Dis. 2007;44(2):287–94. Epub 2006/12/19. CID40300 [pii] 10.1086/510488 . 17173233

[8] Miller AC, Gelmanova IY, Keshavjee S, Atwood S, Yanova G, Mishustin S, et al Alcohol use and the management of multidrug-resistant tuberculosis in Tomsk, Russian Federation. Int J Tuberc Lung Dis. 2012;16(7):891–6. Epub 2012/04/18. ijtld110795 [pii] 10.5588/ijtld.11.0795 . 22507895 PMC8324013

[9] Ruchkina EV, Beliaeva VV, Pokrovskii VV. [Alcohol abuse in HIV infection]. Ter Arkh. 1996;68(11):51–3. Epub 1996/01/01. . 9045381

[10] Global HIV/AIDS Response: Epidemic update and health sector progress towards Universal Access: progress report 2011 Geneva, Switzerland: World Health Organization UNAIDS UNICEF, 2011.

[11] Leon DA, Chenet L, Shkolnikov VM, Zakharov S, Shapiro J, Rakhmanova G, et al Huge variation in Russian mortality rates 1984–94: artefact, alcohol, or what? Lancet. 1997;350(9075):383–8. Epub 1997/08/09. S0140-6736(97)03360-6 [pii] 10.1016/S0140-6736(97)03360-6 . 9259651

[12] Rhodes T, Sarang A, Bobrik A, Bobkov E, Platt L. HIV transmission and HIV prevention associated with injecting drug use in the Russian Federation. Int J Drug Policy. 2004;15(1):1–16.

[13] Key Facts on HIV Epidemic in Russian Federation and Progress in 2011. In: Organization WH, editor. Geneva: World Health Organization; 2013.

[14] Vasquez C, Lioznov D, Nikolaenko S, Yatsishin S, Lesnikova D, Cox D, et al Gender disparities in HIV risk behavior and access to health care in St. Petersburg, Russia. AIDS Patient Care STDS. 2013;27(5):304–10. Epub 2013/05/09. 10.1089/apc.2013.0019 . 23651108

[15] Krupitsky EM, Zvartau EE, Lioznov DA, Tsoy MV, Egorova VY, Belyaeva TV, et al Co-morbidity of infectious and addictive diseases in St. Petersburg and the Leningrad Region, Russia. Eur Addict Res. 2006;12(1):12–9. Epub 2005/12/15. 88578 [pii] 10.1159/000088578 . 16352898

[16] Samet JH, Krupitsky EM, Cheng DM, Raj A, Egorova VY, Levenson S, et al Mitigating risky sexual behaviors among Russian narcology hospital patients: the PREVENT (Partnership to Reduce the Epidemic Via Engagement in Narcology Treatment) randomized controlled trial. Addiction. 2008;103(9):1474–83. 10.1111/j.1360-0443.2008.02251.x 18636998 PMC2588416

[17] Mathew TA, Yanov SA, Mazitov R, Mishustin SP, Strelis AK, Yanova GV, et al Integration of alcohol use disorders identification and management in the tuberculosis programme in Tomsk Oblast, Russia. Eur J Public Health. 2009;19(1):16–8. Epub 2008/12/30. ckn093 [pii] 10.1093/eurpub/ckn093 19112073 PMC2720714

[18] Shin SS, Mathew TA, Yanova GV, Fitzmaurice GM, Livchits V, Yanov SA, et al Alcohol consumption among men and women with tuberculosis in Tomsk, Russia. Cent Eur J Public Health. 2010;18(3):132–8. Epub 2010/11/03. 21033607 10.21101/cejph.a3590 PMC3062936

[19] World Health Organization. Composite International Diagnostic Interview 1.1. Geneva, Switzerland: 1993.

[20] Shin S, Livchits V, Connery HS, Shields A, Yanov S, Yanova G, et al Effectiveness of alcohol treatment interventions integrated into routine tuberculosis care in Tomsk, Russia. Addiction. 2013;108(8):1387–96. Epub 2013/03/16. 10.1111/add.12148 23490304 PMC3710528

[21] Kladov SY, Konobeyevskaya IN, Karpov RS. Alcohol and premature death rates of the population of Tomsk Region. Bull Siberian Med. 2010;1:126–9.

[22] Saunders JB, Aasland OG, Babor TF, de la Fuente JR, Grant M. Development of the Alcohol Use Disorders Identification Test (AUDIT): WHO Collaborative Project on Early Detection of Persons with Harmful Alcohol Consumption—II. Addiction. 1993;88(6):791–804. Epub 1993/06/01. . 8329970 10.1111/j.1360-0443.1993.tb02093.x

[23] Weissman MM, Sholomskas D, Pottenger M, Prusoff BA, Locke BZ. Assessing depressive symptoms in five psychiatric populations: a validation study. Am J Epidemiol. 1977;106(3):203–14. Epub 1977/09/01. . 900119 10.1093/oxfordjournals.aje.a112455

[24] Sobell LC, Sobell MB. Timeline follow-back: a technique for assessing self-reported alcohol consumption In: Litten R, Allen J, editors. Measuring Alcohol Consumption—Psychosocial and Biochemical Methods. Totowa, N.J.: Humana Press; 1992 p. 41–71.

[25] Navaline HA, Snider EC, Petro CJ, Tobin D, Metzger D, Alterman AI, et al Preparations for AIDS vaccine trials. An automated version of the Risk Assessment Battery (RAB): enhancing the assessment of risk behaviors. AIDS Res Hum Retroviruses. 1994;10 Suppl 2:S281–3. Epub 1994/01/01. . 7865319

[26] McLellan AT, Kushner H, Metzger D, Peters R, Smith I, Grissom G, et al The Fifth Edition of the Addiction Severity Index. J Subst Abuse Treat. 1992;9(3):199–213. Epub 1992/01/01. . 1334156 10.1016/0740-5472(92)90062-s

[27] World Health Organization. Geneva, Switzerland: World Health Organization; 2014 Global status report on alcohol and health 2014.

[28] Wilsnack RW, Vogeltanz ND, Wilsnack SC, Harris TR, Ahlstrom S, Bondy S, et al Gender differences in alcohol consumption and adverse drinking consequences: cross-cultural patterns. Addiction. 2000;95(2):251–65. Epub 2000/03/21. . 10723854 10.1046/j.1360-0443.2000.95225112.x

[29] Zaridze D, Lewington S, Boroda A, Scelo G, Karpov R, Lazarev A, et al Alcohol and mortality in Russia: prospective observational study of 151,000 adults. Lancet. 2014;383(9927):1465–73. Epub 2014/02/04. S0140-6736(13)62247-3 [pii] 10.1016/S0140-6736(13)62247-3 24486187 PMC4007591

[30] Fleming MF, Krupitsky E, Tsoy M, Zvartau E, Brazhenko N, Jakubowiak W, et al Alcohol and drug use disorders, HIV status and drug resistance in a sample of Russian TB patients. Int J Tuberc Lung Dis. 2006;10(5):565–70. Epub 2006/05/18. 16704041 PMC1570181

[31] Ehrenstein V, Horton NJ, Samet JH. Inconsistent condom use among HIV-infected patients with alcohol problems. Drug Alcohol Depend. 2004;73(2):159–66. Epub 2004/01/17. S0376871603002886 [pii]. . 14725955 10.1016/j.drugalcdep.2003.10.011

[32] Weinhardt LS, Carey MP. Does alcohol lead to sexual risk behavior? Findings from event-level research. Annu Rev Sex Res. 2000;11:125–57. Epub 2001/05/16. 11351830 PMC2426779

[33] Crooks D, Tsui J, Anderson B, Dossabhoy S, Herman D, Liebschutz JM, et al Differential Risk Factors for HIV Drug and Sex Risk-Taking Among Non-treatment-seeking Hospitalized Injection Drug Users. AIDS Behav. 2014 Epub 2014/07/27. 10.1007/s10461-014-0754-7 . 25063229 PMC4861062

[34] Stickley A, Koyanagi A, Koposov R, Razvodovsky Y, Ruchkin V. Adolescent binge drinking and risky health behaviours: findings from northern Russia. Drug Alcohol Depend. 2013;133(3):838–44. Epub 2013/10/02. S0376-8716(13)00360-8 [pii] 10.1016/j.drugalcdep.2013.08.028 . 24080314

[35] Metzger DS, Woody GE, McLellan AT, O'Brien CP, Druley P, Navaline H, et al Human immunodeficiency virus seroconversion among intravenous drug users in- and out-of-treatment: an 18-month prospective follow-up. J Acquir Immune Defic Syndr. 1993;6(9):1049–56. Epub 1993/09/01. . 8340896

[36] Shuter J, Alpert PL, DeShaw MG, Greenberg B, Chang CJ, Klein RS. Gender differences in HIV risk behaviors in an adult emergency department in New York City. J Urban Health. 1999;76(2):237–46. Epub 2000/08/03. 10.1007/BF02344679 10924033 PMC3455987

[37] Brooks A, Meade CS, Potter JS, Lokhnygina Y, Calsyn DA, Greenfield SF. Gender differences in the rates and correlates of HIV risk behaviors among drug abusers. Subst Use Misuse. 2010;45(14):2444–69. Epub 2010/06/12. 10.3109/10826084.2010.490928 20536356 PMC3169437

[38] Blume SB, Russell M. Sexuality and stigma: the alcoholic woman. Alcohol Health and Research World. 1991;15:136–46.

[39] Laatikainen T, Alho H, Vartiainen E, Jousilahti P, Sillanaukee P, Puska P. Self-reported alcohol consumption and association to carbohydrate-deficient transferrin and gamma-glutamyltransferase in a random sample of the general population in the Republic of Karelia, Russia and in North Karelia, Finland. Alcohol Alcohol. 2002;37(3):282–8. Epub 2002/05/11. . 12003919 10.1093/alcalc/37.3.282

